# Author's Reply

**DOI:** 10.1371/journal.pmed.0020326

**Published:** 2005-09-27

**Authors:** Meir Stampfer

**Affiliations:** **1**Harvard School of Public HealthBoston, MassachusettsUnited States of America

Williamson states that, in my Perspective [1], I erred in pointing out that Sørensen et al. [2] differentiated only between current smokers and nonsmokers. As Williamson notes in his letter [3], the point raised is precisely the analysis presented by the authors. Adequate treatment for cigarette smoking is crucial. Williamson relies on a computer simulation to suggest that treatment is not important, but the plain facts demonstrate otherwise. Computer simulations are, of course, totally dependent on the underlying assumptions. Ample empirical data, coupled with strong biological knowledge, reinforce the importance of smoking as a confounding factor in studies of body weight and mortality. For example, in our own analysis, the link between overweight and mortality risk was substantially obscured by cigarette smoking, and emerged clearly when never smokers were analyzed separately [4]. The reasons for this are simple. Cigarette smoking is associated in many populations with a lower body mass index, and with higher mortality rates. Moreover, cigarette smoking causes several adverse health conditions that lead to lower body weight and higher mortality risk, such as chronic pulmonary disease and congestive heart failure. Individuals may often live with these conditions for many years, so that lagged analyses (conducted by Sørensen et al., as pointed out by Williamson) that exclude early mortality, though useful, are insufficient by themselves to deal fully with this problem.

Williamson appears to miss the most important point. This is simply not a study of the consequences of intentional weight loss, and can be illustrated by way of a quiz (see Table 1): using data from the Sørensen paper, can the reader guess which is the intentional weight loss group?

**Table 1:**
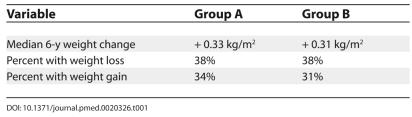
Data from Sørensen et al. [2]

Group A declared an intention to lose weight, but the actual weight changes in the two groups were virtually indistinguishable. Do Williamson and Sorensen et al. seriously entertain the hypothesis that this difference in weight change caused an 88% increase in all-cause mortality rate? Clearly individuals declaring intent to lose weight differ from those who do not. However, it seems implausible to attribute the differences in mortality rate to the tiny differences in weight change.
